# FOXP1 Interacts with MyoD to Repress its Transcription and Myoblast Conversion

**Published:** 2021

**Authors:** Woodring E. Wright, Chuan Li, Chang-xue Zheng, Haley O. Tucker

**Affiliations:** 1 Department of Cell Biology, UT Southwestern Medical School, Dallas TX 75235, USA; 2 Department of Microbiology, University of Texas Southwestern Medical Center, Dallas TX 75235, USA; 3 Department of Molecular Biosciences, the University of Texas at Austin, Austin TX 78712, USA

**Keywords:** Transcriptional regulation, Myogenic regulatory factors, Forkhead box P1, Myoblast differentiation

## Abstract

Forkhead transcription factors (TFs) often dimerize outside their extensive family, whereas bHLH transcription factors typically dimerize with E12/E47. Based on structural similarities, we predicted that a member of the former, Forkhead Box P1 (FOXP1), might heterodimerize with a member of the latter, MYOD1 (MyoD). Data shown here support this hypothesis and further demonstrate the specificity of this forkhead/myogenic interaction among other myogenic regulatory factors. We found that FOXP1-MyoD heterodimerization compromises the ability of MyoD to bind to E-boxes and to transactivate E box- containing promoters. We observed that FOXP1 is required for the full ability of MyoD to convert fibroblasts into myotubules. We provide a model in which FOXP1 displaces ID and E12/E47 to repress MyoD during the proliferative phase of myoblast differentiation. These data identify FOXP1 as a hitherto unsuspected transcriptional repressor of MyoD. We suggest that isolation of paired E-box and forkhead sites within 1 turn helical spacings provides potential for cooperative interactions among heretofore distinct classes of transcription factors.

## Introduction

The five p1 members of the ~100 forkhead (Fkh) transcription factor (TF) family function primarily as transcriptional repressors by employing a highly conserved Forkhead (Fkh) domain to bind DNA with high specificity following homodimerization [1–4]. One such member, Forkhead Box P1 [FOXP1], typically expressed as multiple isoforms [5], is necessary for the proper development of the heart, lung and brain of mammals [6–9]. Previous studies have shown that FOXP1 is essential to various aspects of cardiac development, including formation of the outflow tract, myocardial proliferation and thinning of the ventricular myocardium [3,10]. FOXP1 exists as a mixture of monomers and dimers [11] and belongs to the P-subfamily of Fkh TFs that also include FOXP2-4 [12]. In addition to its Fkh DNA-binding domain, FOXP1 contains a glutamine-rich region, a zinc finger and a leucine zipper required for homodimerization.

Another family of dimerizing TFs critical for heart development and other cellular systems are characterized by their basic helix-loop-helix (bHLH) DNA binding domains. These include four highly conserved myogenic regulatory factors (MRFs) [13,14]. Muscle-specific bHLH TFs cooperate with the MEF2 family of MADS box TFs to activate transcription of muscle structural genes through E-box and MEF2 promoter sites, respectively [13,14]. One of these, Myoblast Determination protein 1 (MYOD1/MyoD), is the topic of this report.

*MyoD1* transcription is largely limited to embryonic somitic precursors and differentiated myogenic cells [13,14]. MyoD functions primarily in development to commit mesoderm progenitor cells to the skeletal myoblast lineage [14] and to continually regulate their status. MyoD also has been shown to regulate muscle repair, as its levels are elevated during skeletal muscle aging [14,15].

*Myod1* transcription is primarily regulated by two enhancer regions [Supplementary Figure 1; 15]. However, regulation through these elements is complex, as combinations of multiple TFs bind to activate or repress in different muscle progenitor cells and phases of differentiation. A range of epigenetic modifications within the 24kb “super enhancer” also contribute to *Myod1* transcription during development and regeneration [15].

FOXP1 and MyoD heterodimerize extensively with both family and non-family members [16]. As an example of the latter, FOXP1 forms cooperative complexes with NFAT2 when bound to the same region of the IL-2 promoter to repress its transcription [17]. MyoD and the other MRFs heterodimerize most prominently with bHLH proteins, including the E2A gene products, E12 and E47 [14,15,18]. Their dimerization leads to increased DNA binding and stimulation of *Myod1* transcription [14,15]. Conversely, ID, which contains a HLH dimerization domain but lacks the basic domain, inhibits MyoD DNA binding by dimerizing and sequestering E2A proteins [14,15,19].

The structure of the forkhead domain consists of a compact packing of three α-helices (H), three β-strands (S), and two loops or “wings” (W), arranged in the order of H1-S1-H2-H3-S2-W1-S3-W2 [20]. Helices H3 and W2 interact with the major and minor grooves of DNA, respectively. We demonstrated [1] that an 84 amino acid segment containing H1 and H2 resembled helices 2 and 1 of MyoD, but not other MRFs, in amphipathicity and in conserved hydrophobic core residues (Supplementary Figure 2). With respect to MyoD, a “basic hook” is formed by a conserved arginine and the curvature of two Prolines. H3 of FOXP1 shares properties with the recognition helix conserved in all MEF homeodomains [1,14,15]. We accurately predicted that a basic region at the C-terminus of FOXP1 provides major DNA contacts [1]. These features were confirmed in the high-resolution structures of both FOXP1 and the highly related FOXA1/HNF-3A complexes with DNA [21–23].

These features led us to hypothesize that FOXP1 might heterodimerize with MyoD. We show here that FOXP1, but not the highly similar FOXA1, heterodimerizes *in vitro* and *in vivo* through its Fkh domain with the bHLH domain of MyoD, but not with several other Class 1 or Class II bHLH domains. We found that this interaction competes with MyoD-E47 heterodimerization, leading to a block in MyoD E-box DNA binding. Through this quenching interaction, FOXP1 represses the ability of MyoD to transactivate E-box-driven promoters in cultured cells or *in vitro*. As a consequence, the ability of MyoD to promote fibroblast conversion to myotubules is impaired by ectopic overexpression of FOXP1. Our results suggest a model for FOXP1-MyoD regulation based on repressor replacement of ID by FOXP1 at the post-proliferative stage of myoblast development.

## Results

### FOXP1 and MyoD are coordinately expressed and interact physically *in vivo*

**Figure 1:**
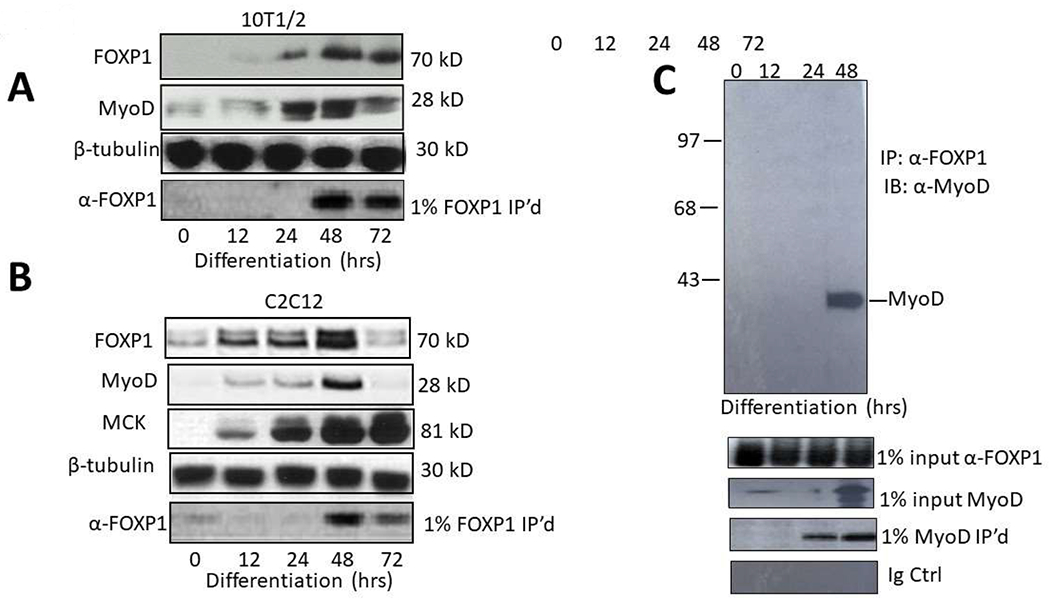
FOXP1 and MyoD are coordinately expressed and interact physically *in vivo*. **A, B.** FOXP1 and MyoD expression peaks at ~3 days following serum withdrawal-mediated differentiation in C2C12 myocytes and or 5-azacytidine-mediated differentiation in 10T1/2 fibroblasts. SDS-PAGE fractionation was followed by anti-MyoD Ab or anti-FOXP1 Ab Western blotting. **C.** FOXP1 and MyoD heterodimerize in differentiated C2C12 myoblasts. Standard co-immunoprecipitation (Co-IP)/Western blotting was performed 3 days following serum withdrawal. Nuclear lysates were immunoprecipitated with anti-FOXPI Ab, fractionated on SDS gels and then blotted with anti-MyoD Ab. IP, immunoprecipitation; WB, Western blotting; Loading controls (CTRL) include 1% input lanes, 1% IP’d lanes, Ig-only, and β- tubulin loading controls. Molecular weights are indicated on the right.

Upon serum deprivation, C2C12 myocytes activate transcription of MyoD and undergo cell cycle arrest following transcription of the Cdk inhibitor, p21, and phosphorylation of pRb [24]. Skeletal muscle differentiation then proceeds through the induction of additional MRFs and fusion of myoblasts into myotubes. C2C12 myocytes and 10T1/2 fibroblasts are well-characterized *in culturo* models of this process [25]. When 10T1/2 cells are treated with 5-azacytidine to induce DNA demethylation, they undergo spontaneous differentiation into myotubes [15]. As shown in Figure 1A, both FOXP1 and MyoD expression in 10T1/2 commences at ~48 in culture following 5-azacytidine addition.

We confirmed and extended these results by employing C2C12 myocytes. As shown in the Western blots of Figure 1B, FOXP1 and MyoD reach expression maxima at approximately the same time, 48 hrs following serum withdrawal. Expression of an E box-containing MyoD target gene, Muscle Creatine Kinase (MCK), continues to increase (the MCK promoter is employed in luciferase experiments detailed below).

We employed a standard co-immunoprecipitation/western blotting approach to test if MyoD and FOXP1 heterodimerize in C2C12 myocytes following serum withdrawal (Figure 1C). Nuclear lysates of differentiated cells were prepared under mild, nonionic detergent conditions, Co-immunoprecipitated (Co- IP’d) with anti-FOXP1 antibody (Ab), fractionated on SDS gels and then blotted with anti-MyoD Ab. As shown in Figure 1C, MyoD was specifically Co-IP’d by FOXP1 at 48 hr—about the same time at which both proteins achieve their maximal expression.

Together these data support the hypothesis that FOXP1 and MyoD are coordinately expressed and interact in differentiated myoblasts.

### *In vitro* heterodimerization specificity of the FOXP1 Fkh and the bHLH domain of MyoD

Helices 1 and 2 of the 84 residue FOXP1 fkh are surprisingly similar to the amphipathic structures of helix-loop-helices (HLH) in total length and hydrophobic core density (Supplementary Figure 2). As pointed out in the Introduction, several invariant/conserved residues within helix 2 of Fkh are shared with helix 1 of both highly and distantly related Fkhs. The similarity becomes more compelling when classes of each domain were compared (Supplementary Figure 3).

A strategy similar to the one above was employed to test the specificity of the FOXP1-MyoD interaction. We performed a series of Co-IPs of FOXP1 along with several potential interaction partners– each tagged with N-terminal HA motifs. These included the 4 MRFs (MYF6, MRF4, MyoG, and MyoD) and their interacting partner, E47. We also analyzed potential FOXP1 interaction with a relatively distant bHLH factor, TAL1, as well as related forkhead protein, FOXA1. HA-FOXP3, a recognized FOXP1 interacting protein whose expression is activated by FOXP1 [26], served as the positive control. Each of the HA-tagged pairs were transcribed and translated in rabbit reticulocyte lysates (detailed in Methods and Materials). Lysates were prepared under non-dissociating conditions for SDS-PAGE fractionation and immunoblotting with anti-HA mAb.

**Figure 2:**
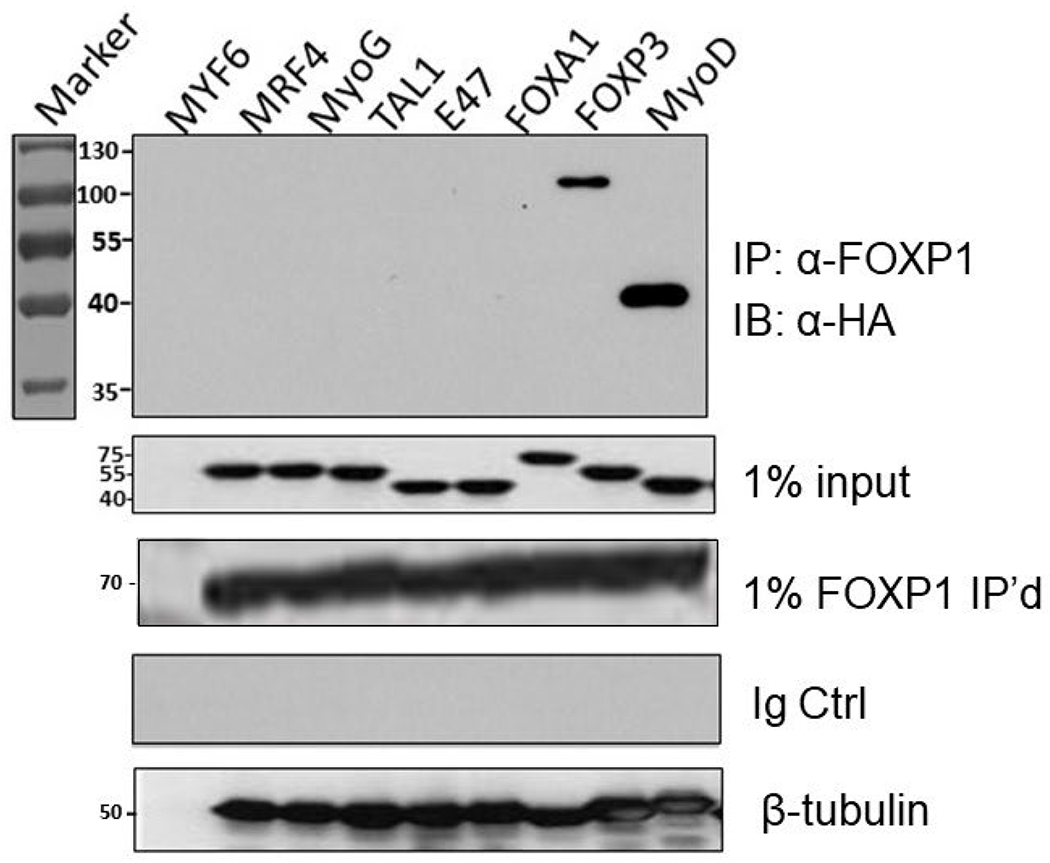
FOXP1 selectively heterodimerizes with MyoD. Putative interaction of MYC-tagged- FOXP1 with several HA-tagged-bHLHs was assessed by Co-IP. These included the 4 HA-tagged MRFs (MYF6, MRF4, MyoG, and MyoD), their interacting partner, HA-E47, as well as the distantly related bHLH factors HA-FOXA1 and HA-TAL1. A previously determined FOXP1 interacting partner, HA-FOXP3 (26) was employed as a positive control. Construct inserts were *in vitro* transcribed and translated within rabbit reticulocyte lysates as detailed in Methods and Materials. Lysates were prepared under non- dissociating conditions for SDS-PAGE fractionation, then Co-IP was performed with MCK-FOXP1 and immunoblotting with anti-HA mAb. FOXP1 associated with FOXP3 and MyoD. Controls: 1% HA-tagged MRF inputs, HA-inputs and Ig-only blots.

As shown in Figure 2 and as anticipated, FOXP1 associated with the positive control, FOXP3. Otherwise, FOXP1 interacted exclusively with MyoD and none of the other bHLH proteins. The specificity of the FOXP1-MyoD interaction was supported by the observation that FOXA1 and FOXP1 share significant conformational and sequence conservation (~90% identity/similarity within their Fkh domains). Curiously and readdressed in Discussion, FOXA1 is a direct activator of MyoD and Pax3/7 in myoblasts [27].

### FOXP1 blocks DNA binding of MyoD homo- and heterodimers with specifically and avidity

**Figure 3:**
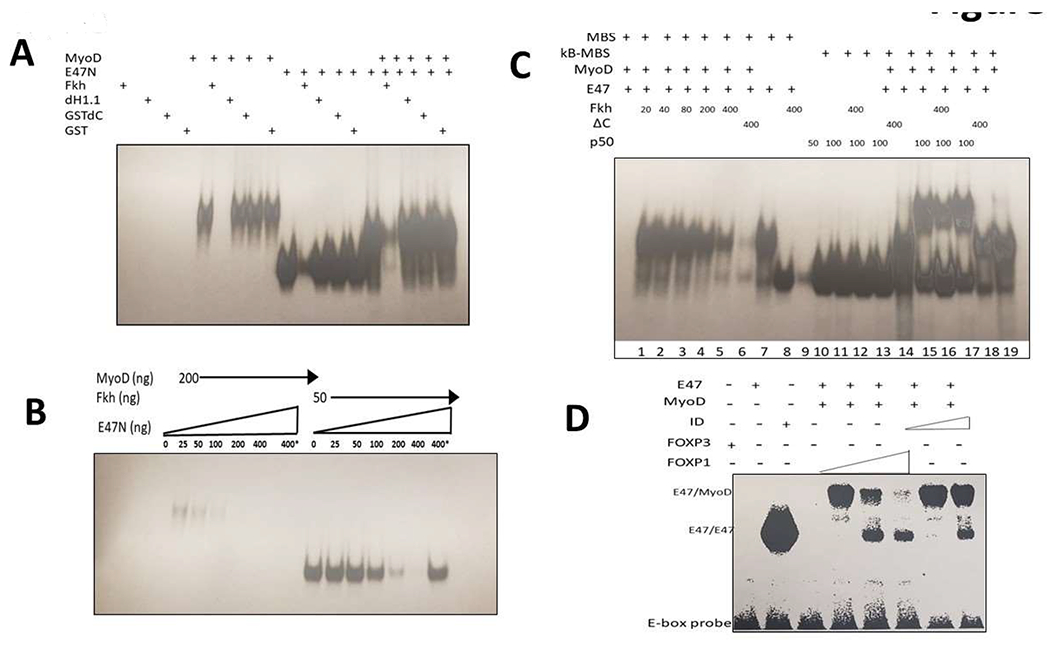
FOXP1 specifically and avidly blocks DNA binding of MyoD homo- and heterodimers. Each of the EMSA experiments were performed with ^32^P-labled DNA or ^32^P-labled oligonucleotide probes. **A.** FOXP1 specifically blocks MyoD homodimer and MyoD-E47 heterodimer binding to the muscle Creatin Kinase (MCK) promoter. EMSA was employed using bacterially expressed MyoD (200ng/reaction), E47N (50ng/reaction) with GST-dH1.2, GST-dC and GST proteins (150ng/each) serving as controls. MyoD-E47 heterodimers were formed prior to addition of the GST-FOXP Forkhead domain (GST-Fkh) which was generated and purified in bacteria. GST-Fkh strongly and specifically inhibited the binding of MyoD homodimers and MyoD-E47 heterodimers. **B.** Titration of GST-Fkh-mediated inhibition of MyoD and MyoD-E47 DNA binding. Using the probe employed in (A), inhibition of DNA binding was determined as a function of GST-Fkh concentration. Proteins were pre-incubated at 37°C for 15 min; MyoD homodimers (200ng/reaction); E47N (50ng/reaction); GST-Fkh concentrations (ng) indicated on Fig. 3B. Full inhibition of MyoD dimers occurs at 100ng, whereas full inhibition of E47N requires 400ng. FOXP1 binds preferentially to homodimers. **C.** Lanes 1-9: The effect of different concentrations of GST-Fkh on MyoD-E47N binding; MyoD (200ng), E47N (50ng), GST-Fkh levels shown on the film. GSTdC added to control lane. Lanes 10-19: An oligonucleotide (oligo) probe containing 1 MyoD and 1 NF-kB binding site (two different binding sites on the same oligo). Lanes 10-13: Bacterially expressed p50(κB) binds specifically to the κB recognition site with no interference with GST-Fkh; Lane 14: MyoD-E47N heterodimers bind to the same oligo. Lanes 15-17: Binding of MyoD-E47N heterodimers and p50 homodimers to the same oligo. The lower complex: p50 binding; the upper complex: double site binding. Lane 16: GST-Fkh inhibits primarily MyoD-E47 complexes (lower intensity of the upper complex; higher intensity of the lower complex represents p50 binding). **D.** Specificity and affinity of FOXP1-MyoD interaction. EMSA performed with an unrelated bHLH TF, FOXA1 and with the MyoD inhibitor, ID1. Full length FOXP1, FOXA1, MyoD, and ID1 were synthesized *in vitro* reticulocyte lysates (detailed in Materials and Methods). Neither FOXA1 nor FOXA1-1 super-shifted complexes formed by FOXP1-MyoD bound to the MCK promoter probe, whereas ID1 super-shifts occurred only at 10-fold higher concentrations.

We cared out a series of EMSA DNA binding experiments to determine the effect of FOXP1 on MyoD homomeric and MyoD-E47 heteromeric DNA binding. Initially we employed a ^32^P-labled probe carrying a single MyoD binding site from the MCK enhancer as probe. GST-linker histone H1.2 (dH1.2), GST-DC and GST-alone served as controls. As shown in Figure 3A, we observed that GST-Fkh strongly and specifically inhibited the binding of MyoD homodimers and MyoD-E47 heterodimers when the dimers were allowed to form prior to addition of the Fkh proteins.

We then titrated DNA binding over several concentrations of GST-Fkh by prior incubation of proteins with the same probe. We observed that full inhibition of MyoD homodimers occurred ~4 times more avidly than that of E47-MyoD monomers. This suggested that FOXP1 binds preferentially to homodimers (Figure 3B). GST-Fkh showed partial resistance when heated to 80°C for 5 minutes prior to initiation of the reaction with probe (Supplementary Figure 4). This suggested that FOXP1 stability is thermo-protected by MyoD association.

Next, we tested the effect of different concentrations of FOXP1 on formation of MyoD-E47 heterodimers; GST-dC served as control (Figure 3C). In lanes 10-19, we employed as probe an oligonucleotide that contains binding sites for MyoD and for NF-κB p50. We observed that in Lanes 10- 13, p50 binds specifically to its DNA recognition site and GST-Fkh does not inhibit its binding. In Lanes 15-17, we observed that binding of MyoD-E47 heterodimers and p50 homodimers to their respective sites occur even when delivered on the same oligonucleotide probe. The lower complex is composed of p50 and the upper complex represents double occupancy. As shown in Lane 16, GST-Fkh primarily inhibits MyoD-E47 complexes, further supporting the specificity of the interaction. The GST-dC mutant in Lane 17 has no inhibitory effect.

As might be expected from both the affinity and the specificity results above, neither *in vitro* translated, full-length FOXA1 nor the unrelated bHLH TF, TAL1, super-shifted complexes formed by FOXP1-MyoD and ^32^P-labeled MCK enhancer (Figure 3D); ID super-shifts required a 10-fold higher concentration.

Taken with the immunoprecipitation data of Figures 1 and 2, these results support our structural- based hypothesis that FOXP1 interacts avidly and selectively both *in vivo* and *in vitro* with MyoD-DNA complexes.

### FOXP1 inhibits MyoD transcription in cultured myoblasts

**Figure 4:**
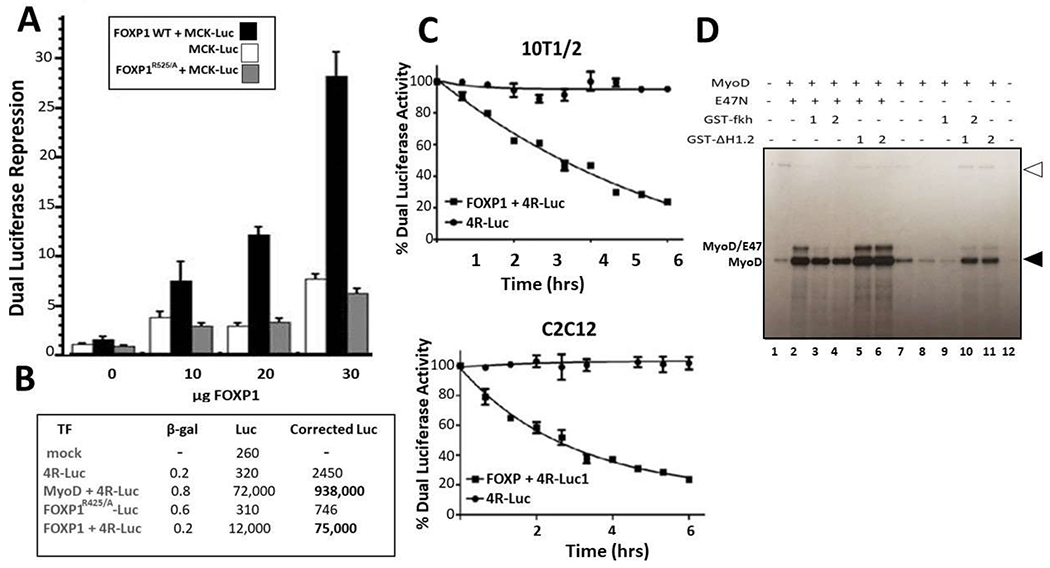
FOXP1 inhibits MyoD transcription in cultured myoblasts and *in vitro.* **A.** FOXP1 repression of MCK E box-containing firefly luciferase expression [29] following 5-azacytidine-mediated differentiation of T101/2 myoblasts. FOXP1 was transfected at varying amounts (indicated on the x-axis as a CMV-based expression plasmid. When normalized to co-transfected *renilla* luciferase controls, ~20- fold FOXP1 repression was observed in cells that received the highest input of FOXP1 (estimated as ~3- fold molar excess over MyoD relative to empty vector control). Negative controls include a FOXP1-DNA binding domain mutant (FOXP1^R525/A^) [30] and luciferase vector alone. **B.** FOXP1 repression of firefly luciferase driven by 2 E-box consensus MyoD binding sites (2R-luc) [32] measured 5 days post induction of C2C12 differentiation. Negative controls included FOXP1^R525/A^. FOXP1 repressed 2R-luc ~12-fold as normalized to co-transfected β-galactosidase expression. **C.** Time course following FOXP1 repression of a MCK-driven E box- luciferase reporter in C2C12 (upper panel) or in 10T1/2 (lower panel). Shown in A-C are the means and standard deviations of a minimum of 4 independent experiments. **D.** FOXP1 represses both basal and MYOD-activated transcription *in vitro*. Employing the methods detailed by Bengal et al. [33] (detailed in S-Methods), basal transcription factors were fractionated from HeLa cells with E47, GST-Fkh, and GST-dH1.2 bacterially expressed and purified on glutathione beads. The Adenovirus Major Late Promoter (Ad MLP) affixed to 6 MyoD E-box binding sites from the MCK enhancer (Ad MLP+6MyoD binding sites) was employed as substrate. MyoD (200 ng/reaction), E47N and GST- dH1.2 (50ng/reaction); GST-Fkh (200 ng/reaction). Filled arrowhead points at transcripts of the control template (Ad MLP); open arrowhead, to transcripts from the test template (Ad MLP+6MyoD).

We first employed transient transfection assays to determine the effect of FOXP1-MyoD interaction on MyoD-dependent transcriptional activation. We analyzed transcription of an E box-containing luciferase reporter in 10T1/2 mouse fibroblasts following 5-azacytidine induction to myoblasts. The luciferase plasmid was driven by the MCK enhancer–an established MyoD target [29]. FOXP1 was provided in varying amounts as a CMV-based expression plasmid. As showed in Figure 4A, an ~20-fold reduction in *firefly* luciferase activity (normalized to *renilla* luciferase control) was achieved in cells that received the highest input of FOXP1 (estimated as ~3-fold molar excess over MyoD relative to empty vector control). Neither a FOXP1-DNA binding domain mutant (FOXP1^R525/A^) [30] nor vector alone generated significant luciferase activities (Figure 4A). This magnitude of repression is similar to that reported previously [31] for ID/MyoD repression at equivalent DNA ratios.

We next utilized as substrate a construct in which expression of luciferase is driven by 2 E-box consensus MyoD binding sites (2R-luc) [32]. Luciferase activity was measured 4 days post induction of C2C12 differentiation. FOXP1 repressed this 2R-luc substrate ~12-fold when normalized to expression of co-transfected β-galactosidase (Figure 4B). DNA binding-deficient FOXP1^R525A^ served as a negative control.

Finally, we performed a time-course in which luciferase activity of the MCK-driven E box- reporter was measured at various times following transfection and serum withdrawal (C2C12) or 5-azacytidine treatment (10T1/2). Corrected values over a course of 6 days were plotted and are shown in Figure 4C. We observed a general correlation with the timing of MyoD myotonic conversion observed in Figures 1A and 1B.

### FOXP1 represses both basal and MYOD-activated transcription *in vitro*

Reporter assays may fail to distinguish between transcriptional initiation and post-transcriptional modifications (eg, transcript half-life, post-transcriptional modification). Thus, we carried out transcription *in vitro* following the general protocol of Bengal et al. [33]. As described previously [33,34 and S-Methods], basal transcription factors were fractionated from HeLa cells. E47, GST-Fkh, and GST-dH1.2 were bacterially expressed and purified on glutathione beads. For template, we employed the Adenovirus Major Late Promoter (Ad MLP) affixed to 6 MyoD E-box binding sites from the MCK enhancer [35]. As shown in Figure 4D, GST-Fkh repressed both MyoD-driven (Lanes 3 and 4) and basal (Lanes 7 and 8) transcription, whereas controls provided no repression.

These results, coupled with those generated in luciferase assays, strongly implicate FOXP1 as a direct transcriptional repressor of *Myod1*. However, we cannot eliminate squelching, which is revisited in Discussion.

### FOXP1 retards the ability of MyoD to catalyze myoblastic differentiation and proliferation

Cultured C2C12 myocytes convert to elongated myoblasts and then to myotubes either following serum withdrawal or when supplied with exogenous MyoD or several other bHLH myogenic regulators (13-16). This conversion can be monitored anatomically and/or by immunostaining with skeletal muscle-specific antibodies.

**Figure 5:**
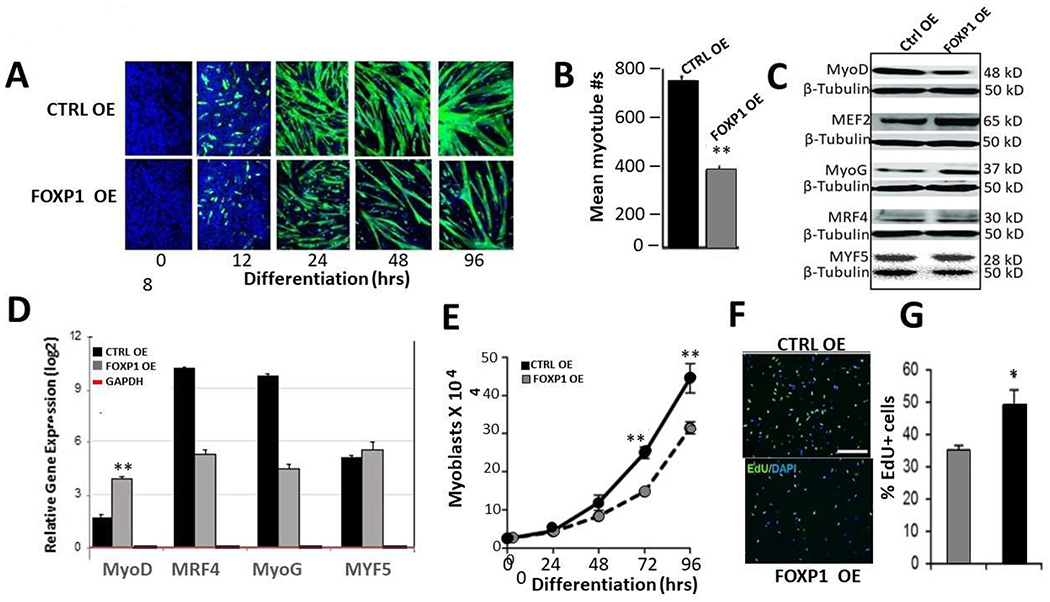
FOXP1 retards MyoD-mediated myoblastic differentiation. **A.** C2C12 myocytes were infected with lentivirus *Foxp1-EF1a-TetRGFP-Bsd*), cultured for several generations (black arrow). At 18 hr following initiation of differentiation (green arrow), Tet^on^ FOXP1 overexpression (OE) was initiated by addition of 1ug/ml doxycycline (dox) as detailed in Materials and Methods. OE kinetics of GFP-illuminated C2C12 cells were measured through day 6. Mock transduced control C2C12 (CTRL) myocytes converted to myoblasts by day 4, whereas FOXP1 OE myoblast conversion was significantly retarded as judged by morphology and GFP fluorescence (green). **B.** Quantification of the data of (A) using epifluorescence microscopy. Shown is the mean of 5 independent measurements (p ≤ 0.05). **C.** Western blot analysis of MRF protein levels of aliquots of FOXP1 OE and CTRL isolated 6 days post C2C12 differentiation of (A). β-tubulin served as a loading control for each Western (individually cut and pasted to make this figure). Molecular weights indicated on the right. **D.** Quantitative analyses of MRF transcripts of (A) isolated at 6 days post lentiviral infection by RT-qPCR. MyoD levels were reduced ~4- fold while other MRFs were either unaffected or insignificantly upregulated. **E-G**. FOXP1 overexpression leads to inhibition of myoblast proliferation. Cell numbers determined by counting **(E)** and proliferative indices determined by EdU/DAPI co-staining **(F)** were both significantly reduced **(G)** following serum withdrawal/retroviral FOXP1 OE as compared to retroviral CTRLs. Experimental details are provided in the text and in Materials and Methods. EdU, 5-ethynyl-2′-deoxyuridine; p ≤ 0.05,*; p ≤ 0.01, **; n=4 (Student’s t tests).

We engineered a tetracycline (Tet)-inducible FOXP1 over-expressing C2C12 cell line via retroviral transduction. Our method is detailed in Materials and Methods and its doxycycline induction kinetics are shown in Supplementary Figure 5. Both the Tet response (R) elements within the promoter and the Tet-controlled transactivator were optimized to achieve lowest background and highest expression (data not shown). Lentiviral-*Foxp1* was infected into C2C12 myocytes, cultured for several generations and then Tet^on^ expression was initiated by addition of doxycycline (dox) 18 hours following serum-deprivation. We then compared the induction kinetics with the Tet^R^ empty vector control over 6 days. As shown in Figure 5A, control C2C12 cell conversion is readily visible by day 4. However, soon after FOXP1 overexpression is activated via dox, we observed significant retardation in the generation of myotubes. Quantification (Figure 5B) of at least 5 independent measurements using epifluorescence microscopy indicated that the reduction was significant (p ≤ 0.05).

Aliquots of control and FOXP1 over-expressing C2C12 cells were analyzed at day 6 following serum withdrawal for accumulation of MRF proteins (Figure 5C). FOXP1 OE led to significant loss of MyoD, whereas MYF5, MyoG, and MRF4 protein levels were relatively unperturbed or slightly elevated. When quantified at the RNA level by RT-qPCR (Figure 5D), MyoD levels were reduced ~4-fold by FOXP1 OE while other MRFs were either unaffected or modestly upregulated (Figure 5D).

During muscle development and growth, quiescent satellite cells are activated to proliferative myoblasts as myogenic progenitor cells. After several rounds of cell division, myoblasts arrest cell cycle and terminally differentiate into mononuclear contractile myocytes. We [7] previously observed that FOXP1 was required for cardiomyocyte proliferation during normal development. Cell numbers of C2C12 FOXP1 OE myoblasts were compared to controls daily following serum withdrawal over a 96-hour time course. As shown in Figure 5E, the number of Ctrl myoblasts were significantly higher than those in which FOXP1 was over-expressed. Next, we quantified their proliferative abilities by measuring DNA synthesis via the incorporation of 5-ethynyl-2′-deoxyuridine (EdU) (Figure 5F). The rate of EdU+ FOXP1 OE myoblasts (35±1.4%) (49.0±4.8%) were significantly reduced (p≤ 0.01) relative to those of controls (50±4.8%) (Figure 5G). These data indicate that high levels of FOXP1 result in reduction of myoblast proliferation and fewer progenies than WT myoblasts.

Collectively our results are consistent with the central hypothesis of this report which holds that FOXP1 is a selective transcriptional repressor of MyoD.

## Discussion

Myoblast differentiation and proliferation are complex events that involve numerous signaling molecules and transcription factors (TFs) (Supplementary Figure 1). The latter include the highly conserved and essential myogenic regulatory factors (MRFs) along with their positive (E12/E47) and negative (ID1-4) heterodimerization partners. In the original cloning and characterization of FOXP1 (then termed QRF1) [1], our structural analysis of the FOXP1 forkhead (Fkh) DNA binding domain arrived at an interesting, yet unexpected, conclusion (Supplementary Figure 2A): That the FOXP1 Fkh domain shared significant linear and tertiary similarity with the bHLH domain of MyoD (Supplementary Figure 2B). Fkh similarity with the other MRFs was far less dramatic. Prompted by these results, we predicted that FOXP1 and MyoD not only interact, but do so with functional consequences. Below we discuss the results generated from this hypothesis and conclude with a model that is predicted by them.

### Embryonic rationale for coordinated expression of FOXP1 and MyoD

MyoD-expressing myoblasts ultimately withdraw from the cell cycle and fuse to form multinucleated myotubes [13–15]. Using fibroblast (10T1/2) and myocyte (C2C12) cell lines that undergo such differentiation following serum withdrawal or 5-azacytidine treatment, we observed quite similar kinetics in the appearance of FOXP1 and MyoD [Figures 1A, and 1B]. This was unanticipated, given that MyoD is expressed exclusively in skeletal muscle and its progenitors [13–15], whereas FOXP1 is expressed far more broadly during adult and embryonic development [1–9]. For example, FOXP1 plays critical roles in development of spinal motor neurons, lymphocytes, bones and connective tissue [6–9].

A potential unifying explanation is that both MyoD and FOXP1 are critical to cardiomyocyte development and function. FOXP is expressed in cardiomyocytes underlying the cushion mesenchyme and in the endocardium [3,6,10]. FOXP1 regulates various aspects of cardiac development, and its loss leads to death and/or complex cardiac phenotypes. In particular, these include defects in outflow tract septation, increased myocardial proliferation, and thinning of ventricular myocardium.

With respect to MyoD, skeletal and cardiac muscle both arise from myogenic mesodermal lineages and share many characteristics [36,37]. The expression patterns of essential TFs and myosin heavy chain (MHC) within ventricular myocardium and skeletal muscle are similar in late embryogenesis [13,38]. These expression patterns become specified as cardiac- or skeletal muscle-only during postnatal development [13,24]. Certain forms of muscular dystrophies associate with cardiomyopathy and chronic cardiac diseases [38]. Another curious observation is that FOXP1 is expressed in both neural crest-derived cells (precursors of bone and tendon) and mesoderm-derived myoblasts [39]. Indeed, fibroblasts can be directly reprogrammed to cardiomyocyte-like cells by introducing fusions of the MyoD transactivation domain [40].

The overlapping pattern of MyoD and FOXP1 during cardiac/skeletal muscle development, along with the common origin of their cardiac/muscle pathologies, suggest an underlying regulatory program for control. While “core” muscle factors such as the MRFs and key TFs (eg, MEF2, GATA and TBX) govern heart development, they primarily contribute to chamber myocardium as opposed to the initial commitment to cardiomyocytes. Thus, understanding the mechanism underlying these initial steps is necessary and will be essential for understanding medical issues relevant to FOXP1, such as cardiomyocyte regeneration [14,15].

### FOXP1 and MyoD heteromerization specificity

The similarity of Helices 1 and 2 of Fkh, with the amphipathic structures of MRFs in length and hydrophobicity (Supplementary Figures 2 and 3), prompted our extension of the MyoD-FOXP1 interaction to the 3 other MRFs (MYF6, MRF4 and MyoG) and their interacting partner (E47). We also tested TAL-1, a distant bHLH protein, as well as the highly similar bHLH, FOXA1. Our Co-IP analyses performed in reticulocyte lysates led to the unexpected finding that FOXP1 interacted only with MyoD and its previously established [23] partner, FOXP3 (Figure 2). These results suggest that the amphipathic helices 1 and 2 of FOXP1 and MyoD are both necessary and sufficient for the interaction.

Since FOXP1 and MyoD each have extensive non-family interactions, we find it useful to speculate how, *in vivo*, their complex might be augmented. MyoD interacts with c-JUN [41], which along with FOS, constitute the NFAT DNA binding and the synergistic NFAT–FOS–JUN–DNA quaternary complex [42]. Initially thought to be T lymphocyte-specific, it is now well established that NFAT proteins direct specific biological programs in a variety of cells and tissues [43]. Both MyoD and FOXP1 interact with the SMAD complex–SMAD3 with FOXP1 in mesendoderm progenitors and SMADs 3 and 5 with MyoD in myoblasts [44–46]. NCOR2, a transcriptional co-repressor that promotes chromatin condensation, interacts not only with FOXP1 but with the SMAD1-4 complex theoretically formed by FOXP1 and MyoD [44–46].

These protein-protein interactions provide the hypothetical macro-complex illustrated in Supplementary Figure 6A. There is no direct evidence of such a complex. However, MyoD and MYF5 contain specific domains that, when aggregated, mediated chromatin remodeling [47]. We speculate that the chromatin structure established by this set of lineage-determining proteins might selectively constrain the activity of other MRFs and add transcriptional specificity to MyoD/FOXP1 heterodimers.

### DNA binding experiments confirm affinity and specificity of the FOXP1-MyoD interaction

EMSA DNA binding experiments not only confirmed but provided additional details of the interaction of the FOXP1 fkh domain with the bHLH of MyoD. First, we observed that if either MyoD homodimers or MyoD-E47 heterodimers were pre-bound to the MCK enhancer, the FOXP1 fkh domain was capable of replacing them (Figure 3A). Full inhibition of MyoD homodimers occurred at ~4-fold lower FOXP1 fkh concentration, plus FOXP1 was thermo-protected by the binding of MyoD (Supplementary Figure 4). These results identified MyoD homodimers as the preferred FOXP1 target and suggested that their association might stabilize their complex from degradation (Figure 3B; Supplementary Figure 4).

Further support for the specificity and affinity of FOXP1 for MyoD was provided in Figure 3D. While neither FOXA1 nor TAL-1 super-shifted complexes of FOXP1-MyoD, FOXP1 super-shifts occurred at 10-fold lower concentrations than required for ID (Figure 3D). Finally, as additional support for specificity, an oligo probe carrying both MyoD and NF-κB p50 binding sites was bound by both TFs, whereas addition of FOXP1 Fkh inhibited only formation of MyoD-E47 dimers (Figure 3C).

These results suggested that FOXP1 might function by increasing the MyoD-E47-dissociation rate or by preventing the bHLH heterodimer from rebinding DNA when they transiently dissociate (readdressed below).

### FOXP1 directly inhibits MyoD transcription in cultured myoblasts

We analyzed the effect of FOXP1 on MyoD transcription initially by transient transfection either 4 days or as a time course following serum withdrawal (C2C12) or 5-azacytidine treatment (10T1/2). We measured the time course of FOXP1 repression of a single E-box-containing luciferase as well as its steady state activity against a 6 E-box luciferase; each were driven by the MCK enhancer (Figures 4A–4C). Overall the magnitude of FOXP1 repression was ~12-30-fold. These results further suggested that maximal repression was achieved near the time of maximal FOXP1 expression during myoblast development (Figures 1A and 1B).

To circumvent caveats associated with luciferase assay interpretation, we measured FOXP1 repression *in vitro* by employing 6 MyoD E-boxes driven by the Adenovirus major late promoter [33]. We observed that FOXP1 strongly repressed both basal and E-box-driven transcription (Figure 4D).

Collectively these results strongly implicate FOXP1 as a direct transcriptional repressor of *Myod1*. However, caution must be applied, particularly regarding the *in vitro* results of Figure 4D. These data do not eliminate the possibility that FOXP1 may interact directly or indirectly with basal transcription factors to prevent their interactions with enhancer/promoter sequences (Supplementary Figure 1); i.e., squelching. This possibility requires further testing. One target of such investigation is the FOXP1-interacting protein, Bromodomain PHD Finger Transcription Factor (BPTF) [48]. As a histone-binding component of the NURF nucleosome- remodeling factor, BPTF catalyzes ATP-dependent nucleosome sliding to facilitate transcription of chromatin.

### Potential mechanism(s) of FOXP1 repression

A central, remaining question is the mechanism by which FOXP1 acts to selectively repress MyoD. In addition to ID1-4, several other MyoD repressors have been identified (Supplementary Figure 1; reviewed by Wardle, [15]). SIM2 is a bHLH-PAS TF expressed in muscle progenitors prior to their migration into the limb [49]. SIM2 appears to prevent entry into the myogenic program via binding to the CE enhancer (Supplementary Figure 1) in embryonic mouse limb buds [49]. DELTEX2 is an E3 ubiquitin ligase that is expressed during adult muscle cell regeneration in myogenic progenitor cells where it inhibits myogenic differentiation [15]. DELTEX2 binds the DRR and PRR regions but not to the CE region of *Myod1*. This leads to an enrichment of the repressive chromatin mark, H3K9me2, likely through inhibiting the lysine demethylase JMJD1C. Myostatin/GDF8, which is produced and released by myocytes acts on muscle autocrine function to inhibit primarily at the level of myoblast proliferation [50]. We find it interesting in the context of our model (Supplementary Figure 6A) that Myostatin inhibition is mediated through SMAD3. However, Myostatin does not share the MyoD specificity of FOXP1, as it inhibits each of the four MRFs [50].

For TFs to direct the activation or repression of gene expression, DNA must be accessible for them to bind. A number of reports have implicated enhancer/promoter accessibility of MyoD enhancer and promoter sequences through multiple epigenetic mechanisms. These include DNA methylation, histone modification and non-coding RNAs (reviewed in [15]).The C/EBP homology protein (CHOP), is expressed in quiescent satellite cells and transiently during myoblast differentiation *in vitro* [15]. Overexpression of CHOP inhibits myogenesis by binding upstream of the MyoD TSS (Supplementary Figure 1). CHOP appears to act via regulating and directly interacting with HDAC1 (51). Using genome-wide approach to identify MyoD modulators, Blum et al. [52] observed requirements for diminished H3K4me1, acetylation of H3K27 (H3K27ac) and reduced recruitment of the H3K4 monomethylase by SET7. In the context of our conjecture above regarding basal transcription factors, MyoD regulatory elements are associated with recruitment of Pol II as well as ncRNAs [15]. Finally, genome-wide analysis led to the identification of TWIST2 as another repressor of myogenic differentiation [53] (Supplementary Figure 1). Knockdown of TWIST2 in Rhabdomyosarcoma cells resulted in up-regulation of MyoD and MyoG as well as a decrease in proliferation.

FOXP1 interacts with several epigenetic modifiers that catalyze repressive marks. These include Metastasis-associated protein (MTA1), a component of the histone-deacetylase multi-protein complex, NuRD [54]. NuRD regulates transcription by modifying the acetylation status of target chromatin. The FOXP1 interacting protein SATB2, on the other hand, binds to DNA at nuclear matrix-associated regions (MARs), which have been shown to induce local chromatin-loop remodeling by recruiting chromatin remodeling or HDAC co-repressors [55]. Of particular interest in the present context is the interaction of FOXP1 with NCOR2/SMRT [56], which bridges our theoretical FOXP1-MyoD super-complex via joint binding with SMADs1-4 (Supplementary Figure 6A). The transcriptional activity of the SMAD2/3:SMAD4 heterotrimer can recruit NCOR2 and possibly other transcriptional repressors (reviewed in [57]).

It will be informative to follow these leads with *Myod1*ChIP experiments to measure the promoter/enhancers occupancy of FOXP1 and its potential co-occupancy with previously mapped factors shown in Supplementary Figure 1.

### FOXP1 functions as a selective repressor of MyoD-mediated myocyte differentiation

When FOXP1 overexpression in C2C12 myocytes was initiated following serum withdrawal-mediated differentiation, significant retardation of myotubes was observed (Figure 5A and Supplementary Figure 5). FOXP1 repression was selective, as we observed no reduction of MYF5, MyoG, or MRF4 accumulation when measured either at the protein or RNA levels (Figures 5B and 5C).

Loss of a number of myogenic regulators as well as epigenetic modifiers have been shown to mediate myocyte differentiation/proliferation defects [15]. A particularly relevant one is FOXA1. We observed no formation of an FOXA1-MyoD heterodimer (Figure 2A). However, Hu et al. [27] demonstrated that FOXA1 was a direct activator of MyoD transcription, and its knockdown led to decreased *in vitro* myocyte differentiation. They further showed that *Foxa1* null mice are reduced in their ability to regenerate muscle [27]. While FOXP1 and FOXA1 share high Fkh sequence similarity, they play opposite roles in MyoD transcription. A potential contributor may be the enormous difference in their abilities to equilibrate between homomeric and heterodimeric states [11]; i.e., domain swapping. Homodimer dissociation of FOXP1 in nearly 1,000 times faster and more favorable than in many other proteins, including FOXA1 [11]. This may allow FOXP1 to maintain the folding stability and cooperativity of Helix3 within the DNA binding Fkh domain.

We further suggest that the kinetics of domain swapping within fkh Helix 3 favors FOXP1-MyoD heteromeric association because it is essential for their interaction (Figure 2B and 2C; Supplementary Figure 2). We find it interesting in this context that mutations affecting domain swapping of FOXP proteins are involved in severe diseases, including the IPEX syndrome in humans [58,59].

### A displacement model for FOXP1 repression at the late stage of MyoD-mediated myogenesis

Our rationale for the work described in this report was based on the following observations:

1) Helices 1 and 2 of FOXP1 resemble helices 2 and 1 of some, but not all bHLH proteins, in amphipathicity and hydrophobic core residues; 2) the best match for the above observation is MyoD; 3) The ID1 MyoD repressor is expressed relatively early and the FOXP1 repressor is expressed relatively late in myogenesis; 4) MyoD, ID and FOXP1 are expressed in muscle-restricted tissues. These observations led us to hypothesize that FOXP1 will displace ID at the later stage of myogenesis to insure continual opportunity for repression; perhaps under conditions of muscle damage.

The data supporting this hypothesis and the additional experimental extensions generated in this report are: 1) FOXP1 heterodimerizes selectively with MyoD and not with other MRFs or bHLH domains; 2) this interaction competes with MyoD homodimerization and/or with MyoD-E47 heterodimerization; 3) through this “quenching interaction, FOXP1 represses the ability of MyoD to transactivate E-box driven promoters; 4) FOXP1 impedes the ability of MyoD to catalyze myocyte to myoblast differentiation.

We have rolled these observations into the model of Supplementary Figure 6B. The model holds that at high serum concentration and/or early in myogenesis, ID is available to repress MyoD by stripping off the E12/E47 activator. Under low ID conditions, MyoD homodimers are available to activate their own gene expression as well as early-stage myogenic proliferation genes. When myoblast proliferation is retarded either by normal conditions (or experimentally by reducing serum concentrations), ID is turned off and MyoD is released from ID. This allows it reassociation with E12 or E47 to reestablish heterodimers that positively transactivate essential differentiation genes (eg, via E-boxes upstream of *Mck*). At later stages of myogenesis, in which proliferation is halted and differentiation proceeds, FOXP1 is expressed. We suggest that it then out-competes/displaces E12/E47 to form a repressive FOXP1-MyoD complex. FOXP1 expression remains available to aid in insuring further MyoD repression as well as for regulation of additional differentiation-specific genes.

This model provides a number of testable opportunities. These include the potential role of FOXP1 in late myoblast proliferative arrest. Supportive mechanistic rationale includes the observations that FOXP1 coordinates cardiomyocyte proliferation [10] as well T-cell quiescence, as defined by reversible cell cycle arrest in the Go phase [60,61].

We also point out that FOXP1 is processed into at least 5 previously characterized isoforms [5]. While our Fkh “only” DNA binding domain is not subject to alternative processing, our full-length experiments utilized only the ~80kD FOXP1 “long” isoform [5], which is the major species expressed in skeletal/cardiac muscle [4,7]. Extending the studies presented here to alternative FOXP1 isoforms might prove useful in deciphering the subtleties of FOXP1 structure with MyoD function.

## Materials and Methods

### Cell cultures

Phoenix A cells were the kind gift of Dr. Gary Nolan. Production of recombinant retroviral constructs and infection of cell lines was performed as detailed in S-Methods and as described at http://www.stanford.edu/group/nolan/protocols/pro_helper_dep.html. Briefly, Phoenix A cells were plated and then transfected with retroviral construct DNA using Fugene6 reagent (Roche). Approximately 48 hours post-transfection, supernatants were selected with 3 μg/ml puromycin and split at 80% confluency.

HeLa cells were grown in Dulbecco’s Modified Eagle Medium (DMEM) supplemented with 10% fetal calf serum, 100 U/ml penicillin and 100 μg/ml streptomycin (growth medium) at 37 °C and 5% CO_2_ in a humid incubator.

### C2C12 myocyte induction

Murine C2C12 myoblasts (American Type Culture Collection) were cultured in growth medium in Dulbecco’s modified Eagle’s medium (DMEM) supplemented with 10% fetal bovine serum (HS; Hyclone; Logan, UT, USA) and 1% penicillin-streptomycin at 37°C with 5% CO2. 95% confluent cells were placed in differentiation medium (DM) consisting of DMEM with 2% horse serum. Multinucleated myotubes were visible 2-3 days of differentiation.

### 10T1/2 fibroblast induction

Murine 10T1/2 fibroblasts (American Type Culture Collection) were treated with 3μM 5-azacytidine. After 24 hr exposure, medium was changed back to growth medium, consisting of Eagle’s basal medium plus 10% fetal bovine serum (FBS). The cultures received fresh medium twice weekly. By day 3, confluent cultures displayed multinuclear myotubes.

### Mammalian expression

pEMSV-MyoD, pGEX-3X-MyoD, and pEMSV-E12 were generous gifts from the H. Weintraub laboratory. Expression vector pCMV-HA-MyoD was previously generated by cloning three hemagglutinin epitope (HA) tags at the amino terminus of the cDNA insert in pcDNA3 (InVitrogen). HA-tagged, full-length coding sequences of MyoG, MRF4 and/or their defined fragments were subcloned into the EcoRI–XbaI sites of pRK5 [62].

pEMSV-MyoD, pEMSV-MyoG pGEX-3X-MyoD, and pEMSV-E12 were published previously [33] and were the kind gifts of Dr. E. Bengal. Expression vector pCMV-HA-MyoD was generated by cloning three hemagglutinin epitope (HA) tags at the amino terminus of the cDNA insert in pcDNA3 (Invitrogen). The MCK-chloramphenicol acetyltransferase (CAT) reporter plasmid (p1256MCK), generously provided by S. Hauschka, contains the mouse MCK promoter-enhancer region [63]. Vector pBK-CMV-FOXP1 was described previously (7). N-terminal HA-tagged, full length bHLH factors were generously provided by Dr. Woodring Wright (UT-SW).

### Lentiviral FOXP1 Tet^on^ overexpression

Full length, murine FOXP1A (long isoform) (NM_001012505.1) was cloned by amplified pcr into pCR4-TOPO (Invitrogen, Carlsbad, CA) and inserted under an inducible suCMV promoter into the lentiviral expression vector, EF1a-TetR(GFP-Bsd; Cat#: LVP1172; GenTarget Inc, San Diego CA.). After packaging as described above, ~90% confluent C2C12 myocytes were infected in DMEM media containing 10X Polybrene. Infections were carried out at a dose of ~100 virus particles per cell for 18 h prior to serum withdrawal-mediated differentiation (Supplementary Figure 5). A GFP-Blasticidin (Fluorescent-Antibiotic) Fusion dual marker under the RSV promoter allows doxycycline induction of green fluorescence, realtime monitoring of lentivirus’ expression.

C2C12 myocytes were cultured at 39.5°C in D-MEM/F-12 medium (Gibco) supplemented with 10% fetal bovine serum, 2% chicken serum (Sigma), penicillin/streptomycin mix, and 10 μM 2-mercaptoethanol (Gibco) in the presence or absence of 1 μg/ml Dox. Growth curves were determined by flow cytometry of C2C12 cells attached to plastic microbeads (07313-5; Polysciences). At various times post-differentiation, cells were fixed for immunofluorescence or harvested for Western blotting and RT-qPCR.

### Immunofluorescence microscopy

Cells were grown on glass coverslips, fixed with 4% formaldehyde and blocked in Phosphate Buffered Saline (PBS)-containing 2% goat serum (Invitrogen), 1% bovine serum albumin (Sigma), 0.1% Tween 20, and 0.05% Triton X-100 for 1 hr at RT. The cells were then incubated with MF20 monoclonal antibody (mAb) against MHC (1:40; DSHB) for 2.5 h and subsequently with an Alexa Fluor 488-conjugated secondary antibody (1:200; Invitrogen) for 1 hr at RT. Mounted cells were incubated with DAPI (4′,6- diamidino-2-phenylindole; Invitrogen) and then subjected to microscopy using a Zeiss Axiovert 200 inverted microscope equipped with a Zeiss AxioCam CCD camera.

### Antibodies, immunoprecipitation and Western blotting

Lysates, generated following transfection (either alone or in combinations) of MRFs, bHLH factors and FOXP1 were prepared under non-dissociating conditions for SDS-PAGE fractionation and immunoblotting with the following antibodies: E2A mouse mAb (epitope corresponding to amino acids 195-208 mapping within a region of E2A conserved between E47 and E12), anti-TAL-1 mouse mAb (sc-3932870) and anti-FOXA1 mouse mAb (A-3,sc-514695 were obtained from Santa Cruz Biotechnology (Santa Cruz, CA). Anti-FOXP1 mAb (FJK-16s) was obtained from eBioscience™ (San Diego, CA). Anti-FOXP1 polyclonal rabbit heteroantisera was generated in house (7). MyoD mAb (Cat #MA5-12902) and MyoD polyclonal rabbit Ab (Cat #PA5-23078) were purchased from Invitrogen/Thermo Fisher Scientific (Waltham, MA); anti-Myogenin mouse mAB (ab187373), anti-MYF6 rabbit polyclonal (ab213681) and anti-MRF4 rabbit polyclonal (ab82842) were obtained from Abcam (Cambridge, MA). Secondary antibodies (horseradish peroxidase [HRP]-conjugated anti-rabbit or anti-mouse) were purchased from Abcam. Filamentous actin (F- actin) was stained with FITC-conjugated phalloidin (Sigma-Aldrich; St. Louis, MO). Nuclei were labeled with 4’-6-diamidino-2-phenylindol (DAPI).

For immunoprecipitation, we employed protein-A immobilized on Sepharose CL-4B (Cat.No. P3391) from Sigma Chemical Company (St. Louis, MO).

Lysates from transfected or untransfected C2C12 and 10T1/2 cells were prepared under non-dissociating conditions for SDS–polyacrylamide gel electrophoresis (SDS-PAGE) fractionation and immunoblotting. Our Western blotting procedure, described previously [4], was performed on 12.5% gels with the above mentioned commercial and home-generated Abs. After electrophoretic transfer of proteins from gels to nitrocellulose membranes, the membranes were blocked with 50 mM Tris-HCl (pH 7.4)–150 mM NaCl–0.05% Tween 20 containing 5% skimmed milk and incubated overnight at 4°C with primary antibodies. After fractionation nitrocellulose membranes, the membranes were blocked with 50 mM Tris-HCl (pH 7.4)–150 mM NaCl–0.05% Tween 20 containing 5% skimmed milk and incubated overnight at 4°C with the following primary antibodies: Anti-MyoD (diluted 1:250), anti-myogenin (diluted 1:500), anti-MRF4(diluted 1:1000), anti-MYF6 (diluted 1:1000, anti-anti-β-tubulin (diluted 1:500). Anti-anti-MRF4 (diluted 1:50), anti- Myosin light chain (MLC1, Sigma; diluted 1:500) and anti-HA (12C, Invitrogen, diluted 1:100). Protein loading was normalized by blotting with either anti-GAPDH (Cat #MA5-15738-D68o) or anti-β-tubulin (Cat #PA5-21416) polyclonal Abs from Invitrogen. Gel loading was normalized to protein concentration.

### Electrophoretic Mobility-Shift Assays (EMSAs)

Probes were labeled with polynucleotide kinase and [γP^32^]ATP (6000 Ci/mmol). The probe was separated from unincorporated [γP^32^]ATP on a Sephadex G-50 spin column. The typical EMSA mixture (20 pl) contained 12.5 mM Tris (pH 7.9), 50 mM KCI, 5 mM MgCl2 7.5% glycerol, 0.1 mM EDTA, 1 mM dithiothreitol, 0.5 pg of poly(dI-dC), different concentrations of the bacterial-synthesized or in vitro translated proteins, and 10-20 fmol of ^32^P-labeled probe. In most cases, 1 pl of whole-cell extract or nuclear extract (10 mg/ml) was added to the reaction. When heterodimeric complexes were formed, the proteins were added before the probe and left to incubate at 37°C for 10 min.

The binding reaction took place at 30°C for 20 min. The reaction mixture was then applied to a 4% nondenaturing polyacrylamide gel in 0.25X TBE (lx TBE = 90 mM Tris/64.6 mM borate/2.5 mM EDTA, pH 8.3) and electrophoresed at 20 mA at 40°C.

In the dissociation-rate experiments, the same binding conditions were applied, but the reactions were scaled-up according to the number of time points that were taken. After 30 min of binding at 30°C, 100 ng of nonradioactive competitor oligonucleotide at each time point was added (200-500 excess over probe DNA). Samples (20 pl) were then taken at different times after the addition of nonradioactive competitor and loaded immediately onto a gel that was running at 40°C.

### Transfections and luciferase Assays

Transient transfections were performed with Fugene6 (Boehringer Mannheim) according to manufacturer’s instructions. C2C12 and 10T½ cells were grown in Dulbecco’s modified Eagle’s medium (DMEM) supplemented with 10% fetal calf serum (GIBCO/BRL). Briefly, 0.3 μg of reporter [4R-tk-luc [64] or MCK4800-luc [65]] and 0.3-12 μg of each activator (EMSV-MyoD, EMSV-FOXP1, EMSV-FOXP1R525A) was mixed with 3 μl of Fugene6 and added to cells in six-well plates. After 24 hr, the medium was changed to differentiation medium (DMEM with 2% horse serum), and 24 to 96 hr later, cells were harvested for dual luciferase [66] and β-galactosidase (β-gal) [67]. Luciferase activities were normalized to β-gal and are reported in comparison to the basal activity of the parental vectors. Samples were processed at the above mentioned time points using passive lysis buffer (Promega Dual Luciferase Reporter assay system, E1910). Firefly luciferase activity was measured (Spectra max, Molecular devices and Varioskan Flash, Thermo Scientific) after adding the respective substrate in the samples. P-values were calculated for 5 assays done in triplicates.

### *In vitro* transcription/translation

bHLH proteins were *in vitro* transcribed and translated in a coupled rabbit reticulocyte lysate using HA-tagged constructs in pEMSVscribe, a plasmid in which the LTR and SV-40 poly(A) addition signal are flanked, respectively, by T3 and T7 promoters (Promega). Plasmids were linearized and *in vitro* translation (l-2 ug RNA 50 ul/) was carried out for 90 min at 30°C using rabbit reticulocyte lysate (Promega). To generate radiolabeled proteins, we employed ^35^S-methionine (>800 mmol; New England Nuclear). Translation reactions were stored at −70°C prior to analysis on SDS-PAGE.

### *In vitro* transcription

*In vitro* transcription was performed according to the method of Bengal et al. [33] and detailed in S-Methods. Briefly, MyoD binding sites (MBS) were inserted into plasmid pML-52/260, which carries the adenovirus type 2 (Ad2) major late promoter (MLP) and a G-less cassette of 260 bp. The clones used for our studies carried six MBS (generated as direct repeats of a 33 bp) and 4 mut MBS (two direct repeats of the ds 33-mer). MyoD proteins were purified by standard procedures, and E47N protein was expressed and purified as described [33]. Partial purification of basal TFs were prepared as described [34]. Transcription factors IIB and IIE (TFIIB and TFIIE) were purified from recombinant E. coli cells [68,69]. TFIIA, TFIIB, TFIID, TFIIF, and TFIIH were purified from 500 ml of HeLa nuclear extract. Steps used to generate the TFIIA, TFIID.TFIIF and TFIIH were previously described [70]. RNA polymerase II was purified essentially as described [71].

Transcription reaction mixtures for crude nuclear extracts are detailed in Bengal et al. [16]. Nuclear extract was added together at the designated amounts of bacterially expressed proteins as described in the legend to Figure 4D. Two DNA templates (control and test) were added to each reaction at 50-250 fmol each. Reaction mixtures were preincubated for 45-60 min at room temperature before nucleotides were added to initiate the reactions. Then nucleotide triphosphates (NTPs) were added (0.5 mM ATP, 0.5 mM CTP, and 15 μM UTP) plus ~10 μCi/reaction of [α-^32^P]UTP (800 Ci/mmol). Reactions were terminated by the addition of RNase T1 (Boehringer Mannheim) at 300C for 60 min, 0.2% SDS and proteinase K (660 ug/ml). After 10 min at 37°C, reaction mixtures were extracted with phenol/chloroform 1:1 (vol/vol), and transcripts were precipitated with ethanol. Reactions were then visualized on SDS-PAGE.

## Supplementary Material

Supplementary Figures and Table

Supplementary Figure Legends and Methods
