# Supplementary material for: FOXP1 Interacts with MyoD to Repress its Transcription and Myoblast Conversion
Source: J Cell Signal. Author manuscript; Available in PMC 2021 Feb 4. (PMC7861563)
Supplement: Supplementary Figures and Table [file NIHMS1660932-supplement-Figures.pdf]

S-Figure 1

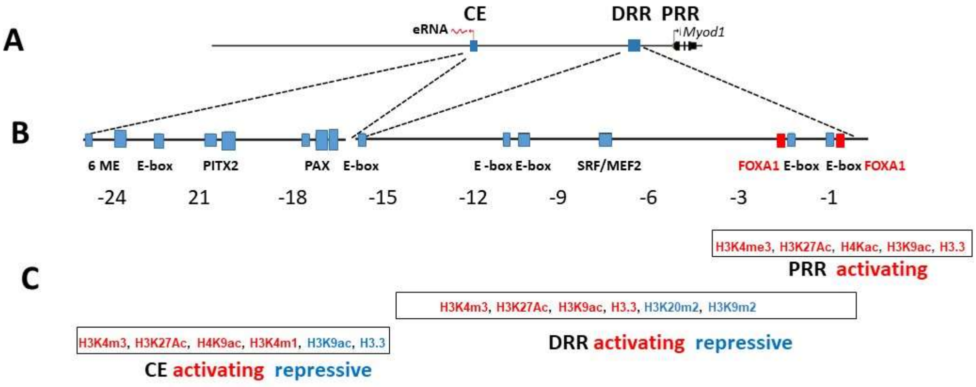

S-Figure 2

A

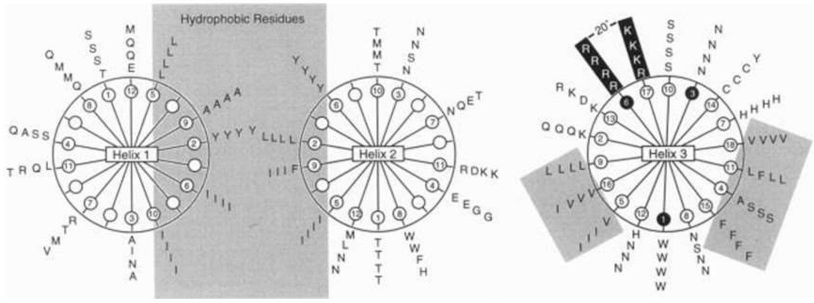

B

	helix 2/1	loop	helix 1/2
**FOXP1**	LNEIYMWFTRM		ASL-IRQAILESPEK-Q
**MyoD**	VNEQFET-LKRC	TSTNPNQRLPK	VSEILRNAIRYI- ESLQ
**Myogenin**	AS	-LL-	HQ R
**MYF5**	Q	T	
**MRF4**	I	VA	SS R

S-Figure 3

Helix 2	LNEIY	Fkh	Class I bHLH (E12, E47)
Helix 1	INEAF	bHLH	Class I bHLH (MyoD, Myog, MYF5, MRF4)
Helix 1	VNEAF	bHLH	Class II bHLH
Helix 2	LNGIY	Fkh	Class VI bHLH (TAL-1)
Helix 1	VNGAF	Fkh	FOXG1, FOXP3, LYL1

S-Figure 4

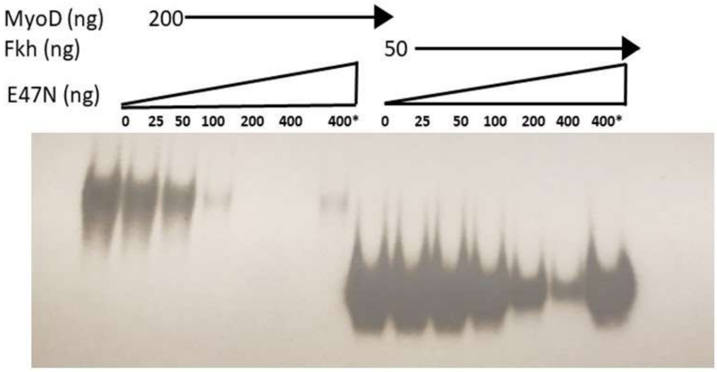

S-Figure 5

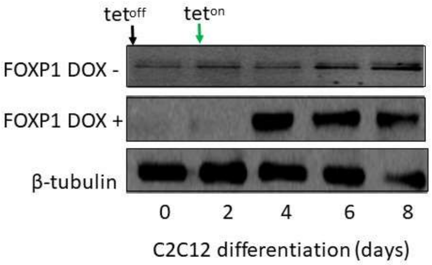

R

S-Figure 6

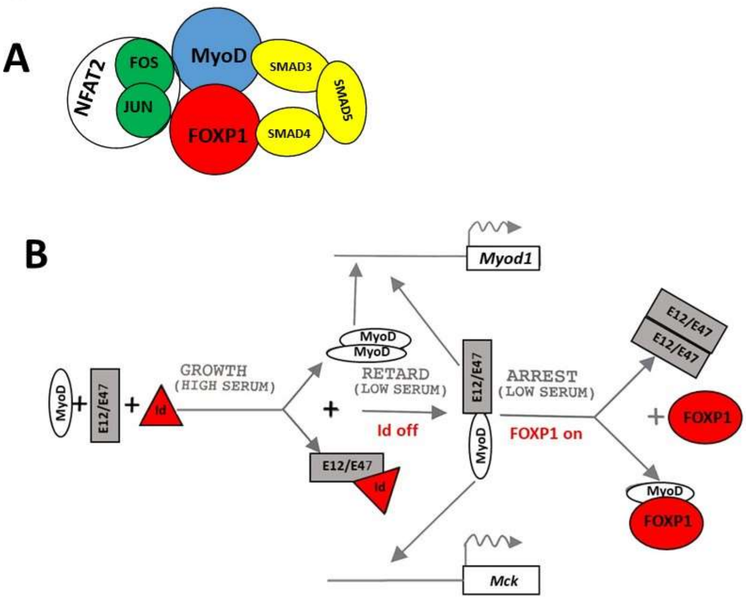

Table 1

Primer	Sequence (5′ → 3′)	Expected cDNA size (bp)
5′ MyHC	TGGTGGTTAAACCAGAGGACG	290
3′ MyHC	GGTAGGCGTTGTCAGAGATGG	
5′ β-Actin	GCATCCTGACCCTGAAGTACC	450
3′ β-Actin	GCTCATAGCTCTTCTCCAGGG	
5′ GAPDH[a]	ATGGTGAAGGTCGGTGTGAACG	1,049
3′ GAPDH	CTCTCTCTTGCTCTCAGTATCC	
5′ MyoD	GAGCAAAGTGAATGAGGCCTT	330
3′ MyoD	CACTGTAGTAGGCGGTGTCGT	
5′ Myogenin	AGTGAATGCAACTCCCACAGC	450
3′ Myogenin	TCAGAAGAGGATGCTCTCTGC	
5′ Id1	ATGAAGGTCGCCAGTGGCAGTG	521
3′ Id1	TCAGCGACACAAGATGCGATCG	
5′ Id2	ATGAAAGCCTTCAGTCCGGTG	404
3′ Id2	TTAGCCACAGAGTACTTTGCT	
5′ Id3	ATGAAGGCGCTGAGCCCG	400
3′ Id3	GTGGCAAAAGCTCCTCTTGTC	
5′ Id4	ATGAAGGCGGTGAGCCCGGTGC	277
3′ Id4	ACCCTGCTTGTTCACGGCGCCG	
5′ β-tubulin	TGGACCGCATCTCTGTGTACT	315
3′ β-tubulin	GCCAAAAGGACCTGAGCGAACA	
5′ FOXP1	CATGCCTCTACCAATGGACAGC	258
3′ FOXP1	GAAGTCGTCACAAACCGCCTCA	
