# Supplementary material for: FOXP1 Interacts with MyoD to Repress its Transcription and Myoblast Conversion
Source: J Cell Signal. Author manuscript; Available in PMC 2021 Feb 4. (PMC7861563)
Supplement: Supplementary Figure Legends and Methods [file NIHMS1660932-supplement-Methods.pdf]

FOXP1 interacts with MyoD to repress its transcription and myoblast conversion

Legends S-Figures and S-Methods

SUPPLEMENTAL (S)-FIGURES

S-Figure 1. Enhancers and transcription factors (TFs) that regulate *Myod* transcription. **A.** Schematic of 50 kb upstream of the *Myod1* gene, including the core enhancer (CE, blue), the distal regulatory region (DRR, blue) and the proximal promoter (PRR, black) upstream of the *Myod1* transcription start site (TSS, black arrow). This "super enhancer" region of ~24kB contains a number of TF binding sites, enhancer RNAs (eRNAs) and epigenetic marks. eRNA is transcribed from the CE and regulates *Myod1* transcription via chromatin accessibility at the *Myod1* promoter. **B**. TF binding sites (blue), that were found either by EMSA or by sequence predictions include: E-boxes which bind to bHLH factors (MyoD and Myf5) as well as to CLOCK, BMAL2, SIM2, MSC; SIX and EYA factors (MEF3 sites), PITX2 (Paired binding site) and for SRF/MEF2 (CaRG box). Binding of FOXA1 to two Forkhead Regulatory Elements (Fkh) are indicted (red) at positions − 940 and −1598 of the PPR. **C.** Activating (red) and repressive (blue) histone marks identified within the *Myod1* PRR, DRR and CE). Adapted from Wardle (15).

S-Figure 2. Helical wheel analysis of the amino acid sequences within helices of FOXP1 and other FOX TF DNA binding forkhead domains. A. Each spoke of is composed of conserved residues from diverse classes of FOX proteins; from inside/outside: FOXP1, FOXA1, FOXG1 and FOXK2. Hydrophobic residues are shaded and the basic face of helix 3 is shown in black. In helices 1 and 2, hydrophobic faces are conserved, whereas the predominantly polar residues (with the exception of T23) are not. The black balls within Helix 3 represent residues that are conserved between the FOXA1 Fkh domain (red bold) and the homeodomain recognition helix (72); modified from Li and Tucker (1). **B.** Amino acid identifies and similarities among FOXP1 and MRFs (MyoD, MyoG, MYF5 and MRF4); dashes (--) inserted to maximize homology. FOXP1 and MyoD are far more similar to one another within Helices 1-2 and 2-1 relative to other MRFs.

S-Figure 3. Similarities among helices 1 and 3 of FOXP1 Fkh, distant Fkhs and bHLH domains. Linear sequence patch identity and similarity (in red) of FOXP1 with distant (FOXG1, FOXP3**)** Fkh domains and with Class 1 bHLH domains (MRFs, TAL1 and LYL1).

S-Figure 4. FOXP1 stability is thermo-protected by MyoD association. A. Validation of purity of GST-fusion proteins employed for EMSA analyses. Fusion of GFP-to the N-terminus of the FOXP1 forkhead (Fkh) and to several proteins utilized in EMSA reactions (including GST-linker histone H1.2, GST-DC and GST alone) is detailed in S-Methods. Briefly Fkh or full length protein domains were cloned into pGEX-6P-2, transformed into E. coli DH5 and purified by standard procedures (33, 34 and detailed in S-Methods) on glutathione Sepharose 4B (Amersham Biosciences). Fusion proteins were mixed with glycerol (to 15%) and DTT (to 2 mM) and stored at −80°C. Purity was assessed by SDS-PAGE with Coomassie blue staining. **B.** Long exposure of Figure 3A demonstrates MyoD-mediated thermo-protection of FOXP1. In Figure 3A, we determined that full inhibition of MyoD homodimers occurs at 100ng of GST-Fkh. Shown here is a longer exposure of Figure 3A. In lanes marked as 400*, 400ng of GST-Fkh was preheated to 80^{0}C for 5 min before mixing it with the other proteins. The retention of Fkh DNA binding indicates a protective effect of MyoD on FOXP1 stability.

S-Figure 5. Lentiviral-mediated FOXP1 overexpression in differentiating C2C12 myocytes. Approximately 90% confluent C2C12 myocytes were infected with ~100 particles/cell of FOXP1-EF1a-TetR(GFP-Bsd) lentiviral particles as detailed in Materials and Methods. Doxycycline (dox) was added (~2ug/cell for 18 hr) prior to day 2 of serum withdrawal-mediated differentiation to initiate lentiviral FOXP1 overexpression (Tet^{On}, downward arrow). Aliquots at each time point were subjected to Western blotting for mock and dox+ expression. β-tubulin served as the loading control.

S-Figure 6. Hypothetical FOXP1 protein complex and mechanism of action during myocyte differentiation. Details of each of the models is elaborated in Discussion. **A.** We speculate that the chromatin structure established by this established set of FOXP1 and MyoD interacting proteins might function as lineage-determining modulators to constrain the activity of other MRFs, thereby providing specificity for MyoD/FOXP1 heterodimers. **B.** A displacement model for FOXP1 control of MyoD transcription and proliferation. We suggest that: (1) Early in myogenesis, ID represses MyoD by stripping off the E12/E47 activator, leaving MyoD homodimers an opportunity to: (2), activate their own as well other stage myogenic proliferation gene expression; (2), once myoblast proliferation is retarded, MyoD is released from ID to re-associate with E12 or E47 to positively transactivate differentiation and proliferation genes; and (4) in early myoblast differentiation, FOXP1 production favorably competes with E12/E47 to form repressive FOXP1-MyoD complexes to insure MyoD repression.

SUPPLEMENTAL (S) METHODS

In vitro transcription. Construction of Plasmids. MyoD binding sites (MBS) were inserted into the pML-52/260 plasmid. This plasmid carries the adenovirus type 2 (Ad2) major late promoter (MLP) sequences (positions -52 to +10). It also contains a cassette of 260 nt that lacks deoxyguanylate residues ("G-less"). The double-stranded (ds) oligonucleotides of the MBS that were used for cloning are as follows, where boldface letters signify MBS: ds 33-mer containing two wild-type (wt) MBS, 5'-GATCCA-GCA-GGT-GT T-GGG-AGG-CAG-CAG-GTGGAG-3' and antisense 3'-GT-CGT-CCA-CAA-CCC-TCCGTC-GTC-CAC-CTC-CTAG-5'; ds 21-mer containing one wt MBS, 5'-GAT-CCA-GCA-GGT-GTG-AAT-TCG-3' and antisense 3'-GT-CGT-CCA-CAC-TTA-AGC-CTAG-5'; and ds 33-mer containing two mutated MBS, 5'-GAT-CCA-GACGGG-TTT-GGG-AGG-CAG-ACG-GGT-GAG-3' and antisense 3'-GT-CTG-CCC-AAA-CCC-TCC-GTC-TGC-CCACTC-CTAG-5'. The ds oligonucleotides were cloned into a Bgl II site positioned immediately upstream of the MLP. Each clone was sequenced to verify orientations and the number of inserts. The clones that were used for further studies carried one MBS (one insert of the ds 21-mer), two MBS (one insert of the ds 33-mer), six MBS (three direct repeats of the ds 33-mer), and 4 mut MBS (two direct repeats of the ds 33-mer). The plasmid that was used as a control in each reaction was pML(C2AT)A50. This plasmid contains essentially the same Ad2 MLP sequence as that in the test template (positions -50 to + 10), and a G-less sequence of390 nt. MyoD was cloned under a phase T7 promoter of the pRK171a vector by creating a Nde I site in the initiator methionine by in vitro mutagenesis. The basic-region Construction of the expression vector for "E47N," an N-terminally truncated version of the HLH protein E47 has been described (1). All of the constructs of bacterially expressing proteins were transformed into BL21(DE3)pLysS Escherichia coli.

TF purification. The MyoD proteins were purified by standard procedures, and E47N protein was expressed and purified as described (2). Partial purification of basal TFs were prepared as described (. HeLa nuclear extracts were prepared as described (3). Transcription factors IIB and IIE (TFIIB and TFIIE) were purified from recombinant E. coli cells as described (4, 5). TFIIA, TFIIB, TFIID, TFIIF, and TFIIH were purified from 500 ml of HeLa nuclear extracts (2.0 x 1011 cells). The phosphocellulose and DEAE-52 chromatographic steps used to generate the TFIIA and TFIID fractions were as described (6). The TFIIB factor was fractionated through two chromatographic steps of phosphocellulose and DEAE-Sephacel as described (7). TFIIF and TFIIH were purified as described (6) up to the DEAE-Sephacel step. The DEAE 5PW and Mono S steps were replaced by a gel filtration step (Superdex 200 HR 16/60, Pharmacia). The Superdex 200 column fractions that contained TFIIF/TFIIH activities in an in vitro transcription assay were pooled and further fractionated on a phenyl-Superose column (HR5/5, Pharmacia) as described (6). TFIIF was eluted between 0.7 and 0.5 M ammonium sulfate and TFIIH was

eluted between 0.2 and 0.1 M ammonium sulfate. Fractions containing TFIIF and TFIIH were separately pooled, dialyzed against buffer C (20) containing 10 mM KCl, and used to reconstitute in vitro transcription reactions. RNA polymerase II was purified essentially as described (6).

Transcription reaction mixtures. For each reaction (40 pl volume) for crude nuclear extracts contained 10 mM Tris (pH 7.9), 7% (vol/vol) glycerol, 50 mM KCl, 0.1 mM EDTA, 6mM MgCl2, 1% PEG, and 5 mM creatine phosphate. Fifty to seventy micrograms of nuclear extracts was added together with the designated amounts in each figure of bacterially expressed proteins. Heterodimers were allowed to form at 370C for 10 min, and then two DNA templates (control and test) were added to each reaction at 50-250 fmol each. Reaction mixtures were preincubated for 45-60 min at room temperature before nucleotides were added to initiate the reactions. The tubes were transferred to 30"C, and 5 min later the NTPs were added to each (0.5 mM ATP, 0.5 mM CTP, and 15 .uM UTP plus 1 p1 per reaction (10 mCi) of [a-^{32}P]UTP (800 Ci/mmol; 1 Ci = 37 GBq). RNase T1 was added at 20 units per reaction (Boehringer Mannheim). Reactions were carried out at 30^{0}C for 60 min and terminated by the addition of 7 mM Tris (pH 7.9), 3 mM EDTA, 0.2% SDS, proteinase K at 660 ug/ml, and 30 pg of tRNA. After 10 min at 370C, reaction mixtures were extracted with phenol/chloroform, 1:1 (vol/vol), and transcripts were precipitated with ethanol.

TF factor addition. The fractionated system contained 12 mM Hepes (pH 7.9), 6 mM MgCl2, 2% PEG, 50 mM KCl, 7% glycerol, 5 mM creatine phosphate, 2 pl of TFIIA (DEAE-cellulose fraction), 2 pl of TFIID (DEAE-cellulose), 1 p1 of recombinant TFIIB (rTFIIB), 2 A1 of recombinant TFIHE (rTFIIE), 2 pl of TFIIF (mono Q), 2 pl of TFIIH (mono Q), and 1 pl of polymerase II (mono Q). In some cases less purified fractions were used: 2 pl TFIID (DEAE-cellulose), 2 pl TFIIE, TFIIF, and TFIIH (DEAE-Sephacel), and 1 pl of TFIIB. Bacterially expressed proteins were added as described in each experiment. Heterodimers of MyoD and E47 were allowed to form at 3^{0}C for 10 min. DNA templates were then added at 50-100 fmol. Activation was optimal between 10 and 20 pmol for WT MyoD, between 2-4 pmol for E47N, and between 1 and 2 pmol of MyoD-E47N heterodimers. The reactions were preincubated at room temperature for 40 min, and transcription was initiated and terminated as described for the crude extracts.

***In vitro* Transcription/Translation.** *In vitro* transcription of MyoD mutants was performed in pEMSVscribe. The LTR and SV-40 poly(A) addition signals of this plasmid are flanked by T3 and T7 promoters, respectively. Wild-type or mutant MyoD plasmids were linearized with BamHI and used in preparative T3 RNA polymerase reactions for Bluescribe plasmids (Stratagene). The 1.3 kb cDNA clone of human El2 in pBS-ATG was previously described (9). Generally, 30-50 pg of RNA was synthesized in a 50 pl reaction using 100 U of T3 RNA polymerase. RNA was purified, ethanol precipitated, and stored at -7O^{O}C in diethylpyrocarbonate-treated distilled water.

For *in vitro* translation, l-2 ug of RNA was used per 50ul reactions for 90 min at 30°C using a pretreated rabbit reticulocyte lysate (Promega). For translations to generate nonradioactively labeled protein, methionine amino acid mixtures minus leucine and minus were each added to the reaction. Parallel translations to generate radioactively labeled proteins were performed using L-35 S-methionine (>800 Ci/mmol (New England Nuclear). Translation reactions were stored at -70°C.

For immunoprecipitation, 5 ul of in vitro translated 35S-labeled proteins was mixed and incubated at 37°C for 20 min. After incubation, 150 ul of buffer (10 mM Tris [pH 7.41, 250 mM NaCI, 5 mM EDTA, 0.25% NP-40) containing 10 ul of anti-MyoD serum was added, followed by 20 ul of packed volume protein A-agarose (Repligen). The samples were shaken for 1 hr at $4O^{0}$C, followed by 3 washes in the same buffer. Samples were then heated to 95°C for 5 min in SDS gel sample buffer, spun, and loaded onto 10-12% discontinuous SDS-PAGE gels. Gels were fixed, treated with 1 M sodium salicylate for 30 min, dried, and fluorographed.

In situ hybridization. Cells grown on 0.01 mg/ml poly-L-lysine (Sigma)-coated coverslips were fixed with 4% paraformaldehyde for 15 min, washed twice with PBST (PBS + 0.5% Tween 20) and permeated with 0.5% Triton X-100 for 25 min. Cells were treated with 2 M HCl for 20 min and then blocked in 1% BSA, 0.1% Triton X-100 in PBS for 1 h at 37 °C. Cell samples were incubated with relevant primary antibodies at various dilutions for 1 h at 37 °C. After extensive washes with PBST, cells were incubated with Dylight 488 or Dylight 549 -conjugated anti-rabbit IgG (Abbkine) or anti-mouse IgG (Abbkine, A23210/A23310). Cells were counterstained with 5 μg/ml 4, 6-diamidino-2-phenylindole (DAPI) in PBS and then mounted onto the slides in anti-fading solution containing 0.25% DABCO. Images were acquired using an Olympus BX51 epifluorescence microscope. Fluorescence intensity was measured with Image-Pro Plus 6.0 Software by manually outlining each nucleus in a given field.

S-References

1. Bengal E, Flores O, Rangarajan PN, Chen A, Weintraub H, Verma IM. Proc Natl Acad Sci U S A. 1994; 91(13):6221-6225.

2. Sun, X.-H. & Baltimore, D. (1991) Cell 64, 459-470.

3. Dignam, D. L., Lebovitz, R. M. & Roeder, R. G. (1983) Nucleic Acids Res. 11, 1475-1485.

4. Ha, I., Lane, W. & Reinberg, D. (1991) Nature (London) 352, 689-695.

5. Peterson et al. (1991) Nature (London) 354, 369-373.

6. Reinberg, D., Horikoshi, M. & Roeder, R. G. (1987) J. Biol. Chem. 262, 3322-3330.

7. Flores, O., Lu, H. & Reinberg, D. (1992) J. Biol. Chem. 267, 2786-2793.

8. Lu, H., Flores, O., Weinmann, R. & Reinberg, D. (1991) Proc. Natl. Acad. Sci. USA 88, 10004- 10008.

9. Murre, C., McCaw, P S., and Baltimore, D. (1989). Cell 56, 777-783.
